# Magnetotail Fast Flow Occurrence Rate and Dawn‐Dusk Asymmetry at X_GSM_ ∼ −60 R_E_

**DOI:** 10.1002/2017JA024776

**Published:** 2018-03-06

**Authors:** S. A. Kiehas, A. Runov, V. Angelopolos, H. Hietala, D. Korovinksiy

**Affiliations:** ^1^ Space Research Institute Austrian Academy of Sciences Graz Austria; ^2^ Institute of Geophysics and Planetary Physics, Department of Earth, Planetary, and Space Sciences University of California Los Angeles CA USA

**Keywords:** magnetotail, ARTEMIS, BBF, fast flows, dawn‐dusk asymmetry, substorms

## Abstract

As a direct result of magnetic reconnection, plasma sheet fast flows act as primary transporter of mass, flux, and energy in the Earth's magnetotail. During the last decades, these flows were mainly studied within *X*_GSM_>−60*R*_*E*_, as observations near or beyond lunar orbit were limited. By using 5 years (2011–2015) of ARTEMIS (Acceleration, Reconnection, Turbulence, and Electrodynamics of the Moons Interaction with the Sun) data, we statistically investigate earthward and tailward flows at around 60 *R*_*E*_ downtail. A significant fraction of fast flows is directed earthward, comprising 43% (*v*_*x*_>400 km/s) to 56% (*v*_*x*_>100 km/s) of all observed flows. This suggests that near‐Earth and midtail reconnection are equally probable of occurring on either side of the ARTEMIS downtail distance. For fast convective flows (*v*_⊥__*x*_>400 km/s), this fraction of earthward flows is reduced to about 29%, which is in line with reconnection as source of these flows and a downtail decreasing Alfvén velocity. More than 60% of tailward convective flows occur in the dusk sector (as opposed to the dawn sector), while earthward convective flows are nearly symmetrically distributed between the two sectors for low AL (>−400 nT) and asymmetrically distributed toward the dusk sector for high AL (<−400 nT). This indicates that the dawn‐dusk asymmetry is more pronounced closer to Earth and moves farther downtail during high geomagnetic activity. This is consistent with similar observations pointing to the asymmetric nature of tail reconnection as the origin of the dawn‐dusk asymmetry of flows and other related observables. We infer that near‐Earth reconnection is preferentially located at dusk, whereas midtail reconnection (*X* >− 60*R*_*E*_) is likely symmetric across the tail during weak substorms and asymmetric toward the dusk sector for strong substorms, as the dawn‐dusk asymmetric nature of reconnection onset in the near‐Earth region progresses downtail.

## Key Points

- Around 43–56% of fast flows near lunar orbit are directed earthward
- The percentage of flows greater than a threshold magnitude that are tailward increases as the threshold increases
- Tailward convective flows and fast earthward convective flows are preferentially found at dusk

## 1 Introduction

Spacecraft observations beyond lunar orbit were rare during the last decades, and consequently plasma flows in the Earth's magnetotail have been studied primarily within *X*_GSM_>−60*R*_*E*_. With this work, we want to shed light on plasma flows near lunar orbit with a 5 year statistical study. Some of the first detailed and comprehensive analyses of plasma sheet flows are given in Angelopoulos et al. (1992) and Baumjohann et al. (1990), who used AMPTE/IRM data in the region *X*_GSM_∼−9 to −19 *R*_*E*_ and showed that fast plasma sheet flows are the main earthward (flux) transport mechanism. Due to the relatively close proximity to Earth, almost no fast tailward flows were observed. Angelopoulos et al. (1994) extended the Active Magnetospheric Particle Tracer Explorers/Ion Release Module (AMPTE/IRM) study to ISEE 2 (apogee *X*_GSM_∼−22*R*_*E*_) data and found only about 20% of all flows being directed tailward. Based on electron flows, observed with ISEE 3, Zwickl et al. (1984) found tailward flows to be dominant throughout the magnetotail (72% (97%) tailward flows for 0 < *X* <− 60(*X* <− 180) *R*_*E*_. Geotail, with an apogee at *X*_GSM_∼−30*R*_*E*_ and several excursions deeper into the tail, allowed investigations farther away from Earth. Nagai et al. (1998) identified tailward and earthward flows within *X*_GSM_∼−50*R*_*E*_ in conjunction with ground station Pi2 pulsations (signifying substorm onset) and found no earthward flows during the 13 Geotail excursions in their database beyond *X*_GSM_∼−30*R*_*E*_, while tailward flows were observed between *X*_GSM_∼−20*R*_*E*_ and *X*_GSM_∼−50*R*_*E*_. They concluded that the average location of the near‐Earth X‐line (the presumed source of these flows) was determined to be between *X*_GSM_∼−20*R*_*E*_ and −30 *R*_*E*_. More recently, Nishimura et al. (2013a, 2013b) related flows observed with ARTEMIS to auroral signatures obtained from the THEMIS all sky imager (ASI) array and found quiet‐time, substorm recovery and steady magnetospheric convection (SMC) flows to be predominantly directed earthward, while tailward flows to occur primarily during the substorm expansion phase. However, due to the requirement of favorable sky conditions for the ASI network, their studies were limited to a few events.

Statistical studies of large data sets have revealed a higher occurrence of plasma sheet flows (e.g., McPherron et al., 2011; Nagai & Machida, 1998; Raj et al., 2002), the X‐line's location (e.g., Eastwood et al., 2010; Genestreti et al., 2014; Nagai et al., 2013), and reconnection‐associated transients, such as traveling compression regions (TCRs, Imber et al., 2011) and dipolarizing flux bundles (DFBs, Liu et al., 2013), in the dusk (premidnight) magnetotail sector. Reviews of these dawn‐dusk asymmetries are given in Runov et al. (2017) and Walsh et al. (2014). Besides magnetotail transients, the current sheet also features a similar asymmetry: Artemyev et al. (2011) and Vasko et al. (2015) showed that the dusk sector current sheet exhibits a smaller thickness, higher current density, and smaller *B*_*z*_ in the dusk sector. From all these observations, one can conclude that there is higher geomagnetic activity in the magnetotail's dusk sector.

Lotko et al. (2014) suggested the meridional gradient in the ionospheric Hall conductivity as source of the magnetotail dawn‐dusk asymmetry. In addition to this ionospheric agent, a magnetotail internal process has been identified by Lu et al. (2016) as a potential contributor to the asymmetry. In their 3‐D global hybrid simulation they observed a stronger Hall effect on the magnetotail's duskside, which they found to be responsible for the larger cross‐tail current density, perpendicular ion temperature, and smaller *B*_*z*_. Such configurations favor reconnection onset (e.g., Sergeev et al., 1993) and result in a higher occurrence of fast plasma flows, TCRs, and DFBs in the dusk sector. The Hall effect and the resultant asymmetry decrease with downtail distance in their simulation. In this paper we investigate the occurrence rate of earthward and tailward flows based on different velocity thresholds and analyze a possible dawn‐dusk asymmetry for both, earthward and tailward flows. We organize this paper as follows: In section 2 we describe how the data was selected. Results on the occurrence rate and flow‐accompanied *B*_*z*_ are presented in sections 3 and 4. Section 5 is dedicated to results on the dawn‐dusk asymmetry; the summary and conclusions are given in section 6.

## 2 Database

**Figure 1:**
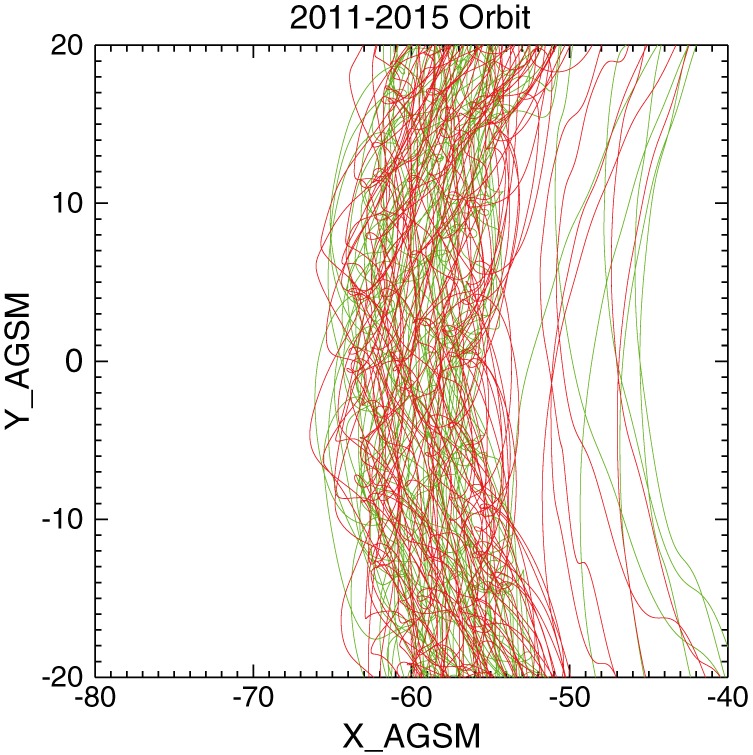
Orbits of ARTEMIS spacecraft P1 (red) and P2 (green) from 2011 to 2015.

The two ARTEMIS probes P1 and P2 were brought into lunar orbits in mid‐2011 with an aposelene of about 19,000 km. Since then the two probes transit the magnetotail for a period of about 4 days every lunar month. Due to the close proximity of the probes and the objective of this study, we utilize observations only from P1 and work with individual data points (spin period samples) rather than flow intervals. We use onboard plasma data from the electrostatic analyzer ESA (McFadden et al., 2008), measuring ion and electron fluxes at energies from about 5 eV to 25 keV and 4 *π* steradian angular resolution once per spin, which varied from about 3 (year 2011) to 4 (year 2015) s. Magnetic field data are provided by the flux gate magnetometer FGM (Auster et al., 2008). Because of a slight time stamp offset between the center times of data collection of the ESA data and the FGM spin fits, we interpolated ESA on FGM data. P1 magnetopause crossings were visually identified based on plasma and magnetic field observations. Consequently, this study covers 58 magnetotail crossings from January 2011 to October 2015. The orbits during this period are shown in Figure 1. To remove mantle, boundary, and magnetosheath flows, we selected samples with *T*_*i*_>300 eV and *n* < 0.5 cm^−3^, typical of plasma sheet but atypical of the other regions.

**Figure 2:**
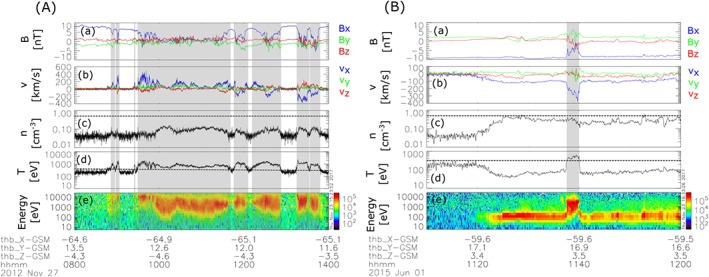
Two examples of P1 observations. From top to bottom: (a) Magnetic field *B*, (b) ion velocity *v*, (c) ion density *n*, (d) ion temperature *T*, and (e) energy spectrograms. The color bar in the bottom panel shows energy fluxes in eV/cm^2^/s/ster/eV. Gray boxes show plasma sheet data points, selected via *T*_*i*_>300 eV (dashed horizontal line in Figure 2d) and *n* < 0.5 cm^−3^ (dashed horizontal line in Figure 2c).

Table 1 lists an overview of the database. Due to the near‐equatorial ARTEMIS orbit, more than 60% of the collected data are plasma sheet samples. Most of the time the plasma sheet was quiet (*v*_*x*_<|100| km/s), leaving about 24% of the plasma sheet samples for this analysis. Figure 2 shows two examples how the plasma sheet is selected. Panel (A) shows a 6 h interval of several plasma sheet encounters with the detection of earthward and tailward flows, including flow reversals. Gray boxes indicate intervals during which above plasma sheet selection criteria (dashed horizontal lines), based on temperature and density, are fulfilled. As can be seen, the selected plasma sheet data points within the gray boxes coincide well with typical plasma sheet energies and flow intervals. The dynamic behavior of the magnetotail, as seen from the magnetic field *B*_*x*_ component, especially between 11:30 and 14:00 UT, makes a selection of flow events difficult, as the probe leaves the plasma sheet for several short intervals (e.g., ∼11:40, ∼12:10, and ∼13:00 UT). If the flows detected in between these lobe excursions are individual flows or part of the same flow, event remains unclear. Consequently, we decided to use individual data points rather than flow events for this study. Panel (B) shows a 50 min interval during which P1 observes tailward flows of cold, dense plasma with a velocity of ∼100 km/s starting at ∼11:20. Based on the energy spectrograms these flows can be identified as mantle plasma flows. At ∼11:40 the probe detects hotter plasma, moving tailward with ∼300 km/s, corresponding to plasma sheet flows, possibly associated with a flux rope as indicated by the bipolar *B*_*z*_ signature and core *B*_*y*_. With our plasma sheet selection criteria, only data points from the plasma sheet flows are selected (gray box) and none from the mantle flow.

**Table 1:** Overview of the Collected Data for this Study

	Data points
All tail	3,392,116
Plasma sheet	2,128,532
Quiet plasma sheet (*v*_*x*_<|100| km/s)	1,622,106
*v*_*x*_>|100| km/s	506,423
*v*_*x*_>|200| km/s	211,432
*v*_*x*_>|300| km/s	92,447
*v*_*x*_>|400| km/s	38,495
*v*_⊥__*x*_>|100| km/s	143,197
*v*_⊥__*x*_>|200| km/s	52,976
*v*_⊥__*x*_>|300| km/s	20,558
*v*_⊥__*x*_>|400| km/s	7,842

## 3 Occurrence Rate

We use the aberrated GSM coordinate system (AGSM). Due to the Earth's orbital motion around the Sun, the radially flowing solar wind aberrates the magnetotail, and hence the noon‐midnight meridian, for about 4° toward *Y*_GSM_ (with an orbital speed of 29 km/s and an average solar wind speed of 400 km/s). Consequently, the AGSM coordinate system is rotated for about 4° in the *X*_GSM_‐*Y*_GSM_ plane relatively to the GSM coordinate system.

**Figure 3:**
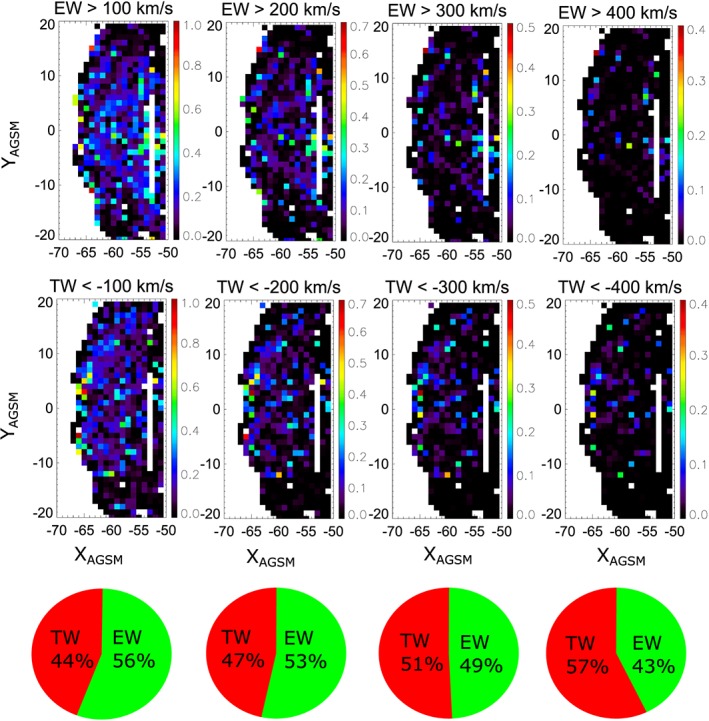
Occurrence rate of (top row) earthward and (middle row) tailward flow data points for different velocity thresholds (columns) and (bottom row) partition of flow direction. The occurrence rate is defined as the number of flow data points in each bin, normalized to all data points P1 collected in the corresponding bin. Only flow data points exceeding the defined thresholds are selected for the corresponding data set. The values in the pie charts show the percentage of flow data points beyond the given threshold which are directed tailward and earthward, respectively.

**Figure 4:**
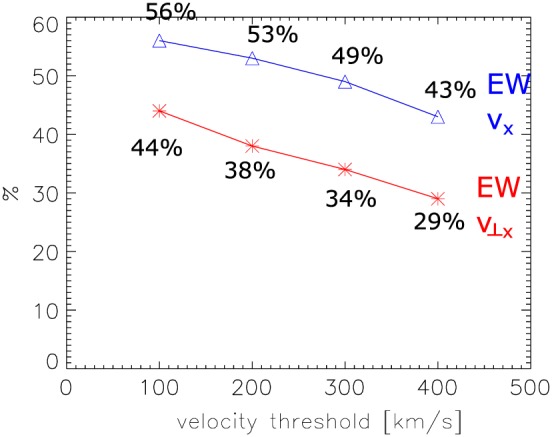
Percentage of earthward flows for different velocity thresholds applied to *v*_*x*_ (blue) and *v*_⊥__*x*_ (red).

In order to determine the spatial occurrence of tailward/earthward flows, we binned the data in 1 ${R_{E}}^{2}$ bins in the *X*_AGSM_–*Y*_AGSM_ plane. We define the occurrence rate as the number of flow data points in each bin, normalized to all data points (flow and no flow) that P1 collected in the corresponding bin. We introduced velocity (*v*_*x*_) thresholds (100, 200, 300, and 400 km/s) in order to resolve any velocity dependence and discriminated between earthward and tailward directed flows. For each velocity threshold, we create a data set, which consists of flow data points exceeding the corresponding threshold. Figure 3 shows the occurrence rate of earthward (top row) and tailward (middle row) flows in the *X*_AGSM_‐*Y*_AGSM_ plane for different velocity (|*v*_*x*_|) thresholds (columns). As can be seen from the bottom row, flows are directed in almost equal parts both tailward and earthward with a dominance of earthward flows of 56% for flows with |*v*_*x*_|>100 km/s and a dominance of tailward flows of 57% for flows with |*v*_*x*_|>400 km/s. For further analysis we decompose the flow vector into components perpendicular (*v*_⊥_) and parallel (*v*_∥_) to the magnetic field. Figure 4 shows the percentage of earthward flows for the same velocity thresholds, which is applied to *v*_*x*_ (blue), corresponding to the values from Figure 3 (bottom row), and *v*_⊥__*x*_ (red). As can be seen, the percentage of earthward flows decreases if only convective (*v*_⊥__*x*_) flows are considered to about 29% (for *v*_⊥__*x*_>400 km/s) to 44% (for *v*_⊥__*x*_>100 km/s). While the percentage of convective (*v*_⊥__*x*_) earthward flows is less than that for all earthward flows (*v*_*x*_), they still contribute significantly to the observed flows at 60*R*_*E*_ downtail and show the same decreasing trend for increasing velocity threshold.

This decrease in the earthward fraction of flows for faster flow speeds is in line with magnetic reconnection as source of these flows and a decrease of the Alfvén velocity with increasing downtail distance to the Earth. The outflow velocity of reconnection is directly proportional to the local Alfvén velocity in the inflow region (Priest & Forbes, 2000), $v_{A}=\frac{B_{0}}{\sqrt{\mu_{0}\;\rho }}$, with *B*_0_, *μ*_0_, and *ρ* as magnetic field in the inflow region, magnetic permeability of free space, and plasma density, respectively. Since the magnetic field strength decreases with downtail distance (Slavin et al., 1985), and plasma sheet plasma mixes with mantle plasma in the deep tail (*X*_GSM_<−150*R*_*E*_, Maezawa & Hori, 1998), increasing the plasma density in the inflow region, the Alfvén velocity, and consequently the outflow velocity of reconnection decreases with downtail distance. Hence, it is expected that the occurrence rate of fast flows originating from the near‐Earth region (corresponding to tailward flows observed with ARTEMIS) is higher than the occurrence rate of fast flows originating from deeper in the tail (corresponding to earthward flows observed with ARTEMIS). This is a potential explanation for the observed decrease (increase) in the earthward (tailward) fraction of flows for faster flow speeds.

## 4 Accompanied *B*_*z*_

**Figure 5:**
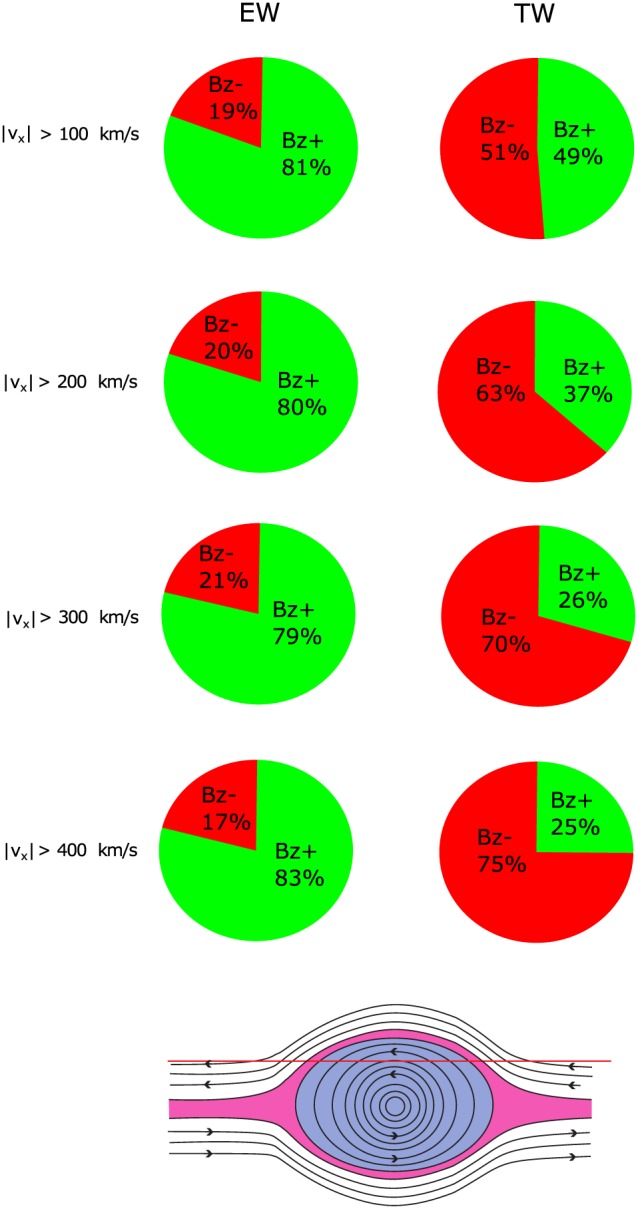
Direction of *B*_*z*_ accompanying (left) earthward and (right) tailward flows for different velocity thresholds. (bottom) Illustration of a spacecraft encounter (red trajectory) with a plasmoid/flux rope.

Figure 5 shows the *B*_*z*_ direction (northward/southward), associated with flow data points for different velocity thresholds. More than 80% of all earthward flow data points are accompanied by positive *B*_*z*_, independent of speed. Conversely, tailward flow data points are predominantly accompanied by negative *B*_*z*_. This would be expected by the nature of magnetic reconnection if it is to be regarded as the source of the flows: Earthward (tailward) flows are accompanied by northward (southward) directed reconnected field lines (e.g., Caan et al., 1979; Kiehas et al., 2009; Nakamura et al., 1994). However, the fraction of tailward flow data points with the expected *B*_*z*_ polarity is lower than the corresponding fraction of earthward flow data points for all thresholds, including the fastest of the flow data points. We attribute this to the presence of tailward moving plasmoids/flux ropes in our database: If a spacecraft encounters a tailward moving plasmoid/flux rope, it will first observe a northward directed *B*_*z*_ in its leading part before it is immersed into its trailing part with southward directed *B*_*z*_. Consequently, a good fraction of tailward flow data points accompanied by positive *B*_*z*_ might be due to encountering leading parts of plasmoids/flux ropes (as illustrated on the bottom of Figure 5).

For increasing flow data point velocity thresholds, the fraction of tailward flow data points accompanied by negative *B*_*z*_ becomes increasingly dominant. Slavin et al. (2003) showed in a superposed epoch analysis that fast flows tend to occur within the trailing part of flux ropes/plasmoids (cf. Figure 9 in Slavin et al., 2003). Since the trailing part of tailward moving flux ropes/plasmoids is accompanied by southward *B*_*z*_, this analysis can explain the higher fraction of tailward flow data points accompanied by southward *B*_*z*_ for data points with higher velocity.

## 5 Dawn‐Dusk Asymmetry

**Figure 6:**
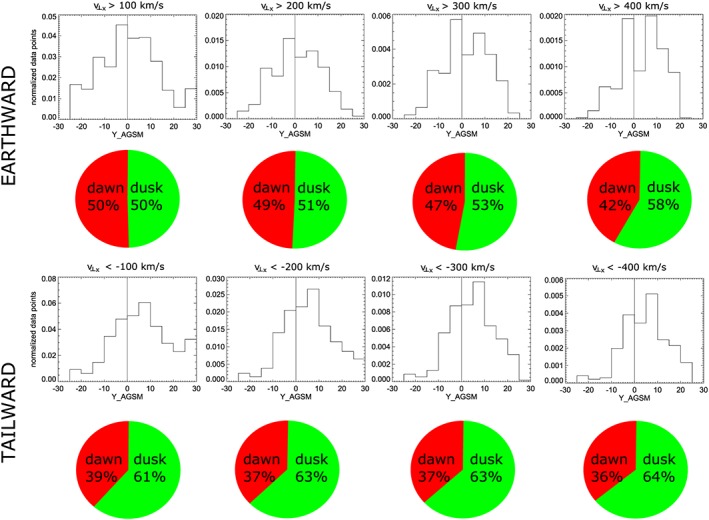
Histograms of flow data points exceeding the velocity threshold as written in the panels for (top row) earthward and (bottom row) tailward convective flows (*v*_⊥__*x*_). Pie charts show the percentage of flows observed in the dawn (red) and dusk (green) sectors.

Figure 6 shows histograms of *v*_⊥__*x*_ flow data points, binned in 5 *R*_*E*_ intervals in *Y*_*AGSM*_ and normalized to the time P1 spent in each bin, integrated along *X*_*AGSM*_ for four different flow speed thresholds for earthward (top row) and tailward (bottom row) flows. Pie charts underneath each histogram show the percentage of flow data points observed in the dawn (red) and dusk (green) sectors. As can be seen, earthward flows do not show a preference to appear either in the dawn or dusk sector. With the exception of flows *v*_⊥__*x*_>400 km/s, the earthward flows are almost evenly distributed over the dawn and dusk sectors. However, the tailward flows show a clear tendency to be observed in the dusk sector, with more than 60% of tailward flows for each speed threshold appearing in the dusk sector.

**Figure 7:**
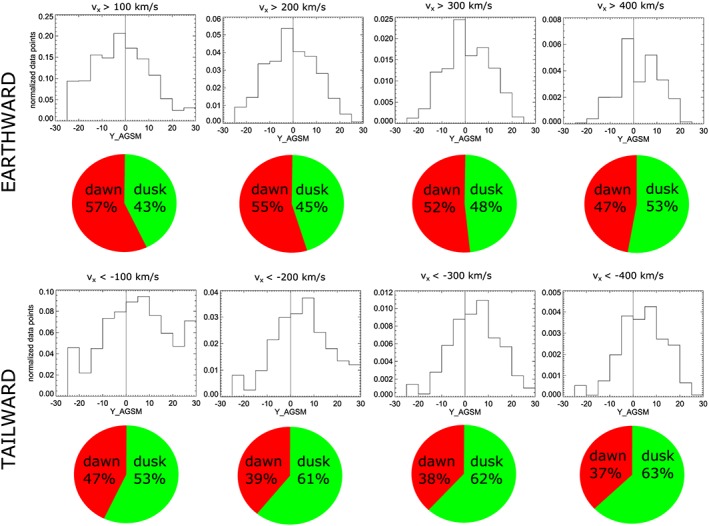
Same as Figure 6 but for *v*_*x*_.

**Figure 8:**
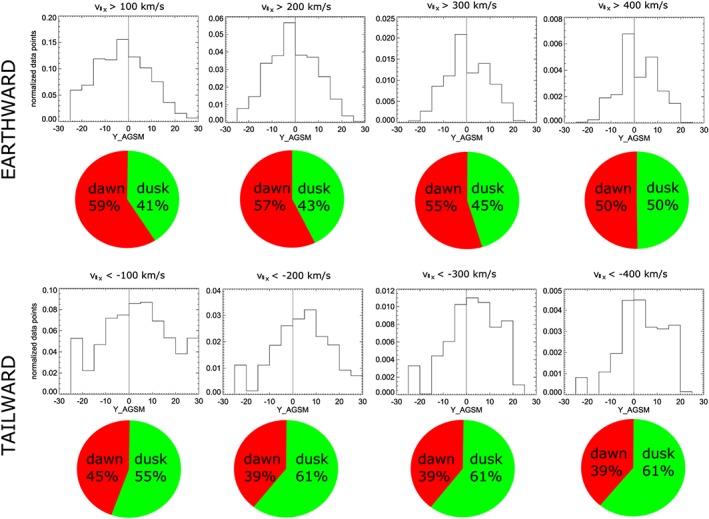
Same as Figure 6 but for *v*_∥__*x*_.

If one considers not just convective flows, but all flows (*v*_*x*_, Figure 7), the situation for tailward flows is similar, while the near‐symmetric appearance of earthward flows is slightly skewed toward the dawn sector. The same is true for parallel flows (Figure 8). These results show that the duskward asymmetry of tailward flows is present at slow tailward flows and dominates at fast tailward flows. The same asymmetry (though less strong) is also present for fast earthward flows.

**Figure 9:**
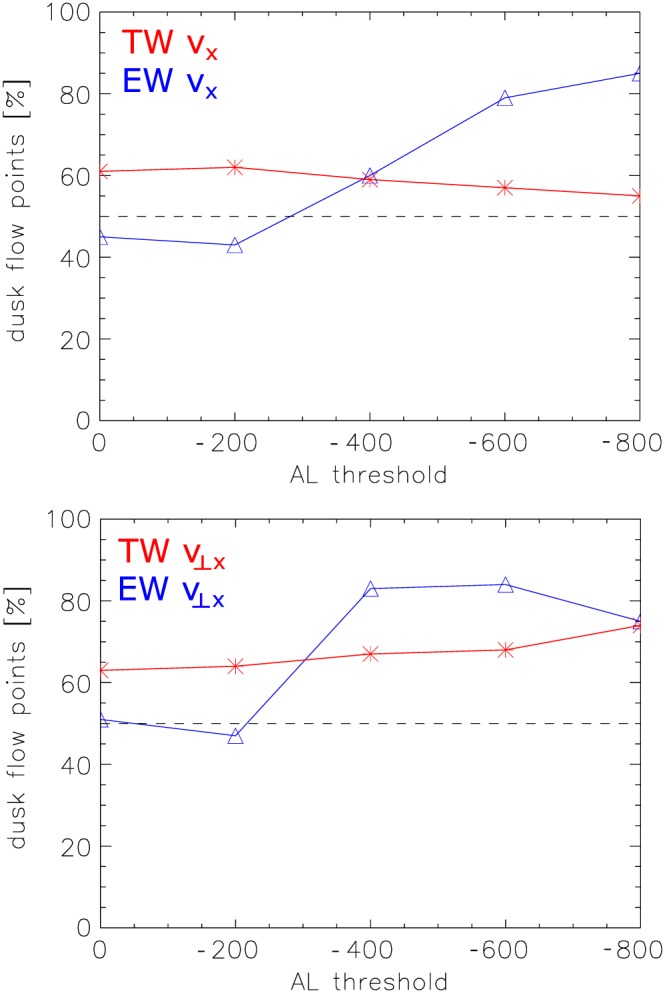
Percentage of flow data points with (top) *v*_*x*_>|200| km/s and (bottom) *v*_⊥__*x*_>|200| km/s observed in the dusk sector relative to AL thresholds.

To account for a possible dependence of the dawn‐dusk asymmetry on geomagnetic activity, we plot in Figure 9 the percentage of flow data points in the dusk sector exceeding |200| km/s relative to AL thresholds for both *v*_*x*_ (top) and *v*_⊥__*x*_ (bottom). For tailward flows (red), the duskward asymmetry is similar for all AL thresholds. However, while earthward flows (blue) are fairly symmetrically distributed over both, dawn and dusk sectors for the lower AL thresholds (AL > 0, AL>|200| nT), they become strongly asymmetric toward the dusk sector for higher geomagnetic activity (AL>|400| nT): 51% of convective earthward flow data points exceeding *v*_⊥__*x*_>200 km/s are observed in the dusk sector for AL > 0 nT, while 83% are observed in the dusk sector for AL>|400| nT. For other velocity thresholds a similar trend can be observed. We interpret these results to mean that near‐Earth reconnection earthward of ARTEMIS and also near but tailward of ARTEMIS occurs asymmetric with respect to the noon‐midnight meridian. However the distant tail reconnection, presumably leading to the slower earthward flows during low geomagnetic activity, occurs symmetric , that is, evenly in the dawn and dusk sectors. For higher geomagnetic activity (AL>|400| nT), a near‐Earth neutral line might retreat downtail, passing by ARTEMIS, which observes its earthward flows. This requires a downtail retreat during high geomagnetic activity of the initial effect which causes the dawn‐dusk asymmetry.

## 6 Summary and Conclusions

We use 5 years of ARTEMIS data to investigate fast flows between about −55 and −65 *R*_*E*_ downtail.

1. A significant portion of earthward directed flows is observed. Fifty‐three percent of the flows exceeding 200 km/s are directed earthward. The fraction of earthward flows decreases with flow speed (flows > 400 km/s: 43% earthward).
2. As expected, earthward flows are predominantly accompanied by northward *B*_*z*_ (about 80% of earthward flows), while tailward flows are accompanied by southward *B*_*z*_ (depending on flow speed between 51% (>|200| km/s) and 75% (>|400| km/s). However, for a given flow threshold fewer tailward flows show the expected reconnection *B*_*z*_ polarity than earthward flows. This discrepancy and the increase in the dominance of southward *B*_*z*_ with tailward flow speed can be explained by the presence of tailward moving flux ropes/plasmoids in our database.
3. Convective flows show a smaller, but still significant fraction of earthward flows (44% (29%) for *v*_⊥__*x*_ flows exceeding |100| (|400|) km/s).
4. A dawn‐dusk asymmetry in the occurrence of flows is visible in the data. Tailward convective flows are observed predominantly in the dusk sector, while earthward convective flows are nearly symmetrically distributed over the dawn and dusk sectors for weak substorms (AL >− 400 nT) and observed predominantly in the dusk sector for strong substorms (AL > 400 nT). Since tailward flows observed by ARTEMIS originate from within *X*_GSM_>−60*R*_*E*_, and earthward flows from deeper in the tail, this indicates that the dawn‐dusk asymmetry is more pronounced closer to Earth and progresses deeper into the tail when substorms evolve beyond AL <− 400 nT. This is in line with recent simulation results identifying the Hall electric field as the dawn‐dusk asymmetry's origin. The results of Lu et al. (2016) show a clear downtail decrease in the strength of the Hall electric field. However, also an ionospheric source of the asymmetry (Lotko et al., 2014) is expected to show stronger effects in the near‐Earth region based on simple mapping considerations.

Our results show an asymmetry not only for convective flows but also for parallel flows, which is in contrast to previous studies (Raj et al., 2002). We attribute this on the presence of ARTEMIS near the reconnection regions and the field line geometry that is unaffected by Earth's dipole. This is contrary to the WIND satellite observations in Raj et al. (2002), that were obtained closer to Earth. The position of ARTEMIS allows prompt arrival of field‐aligned beams to the satellites without significant interaction with the strong Earth field that would otherwise deflect or perturb these beams.

**Figure 10:**
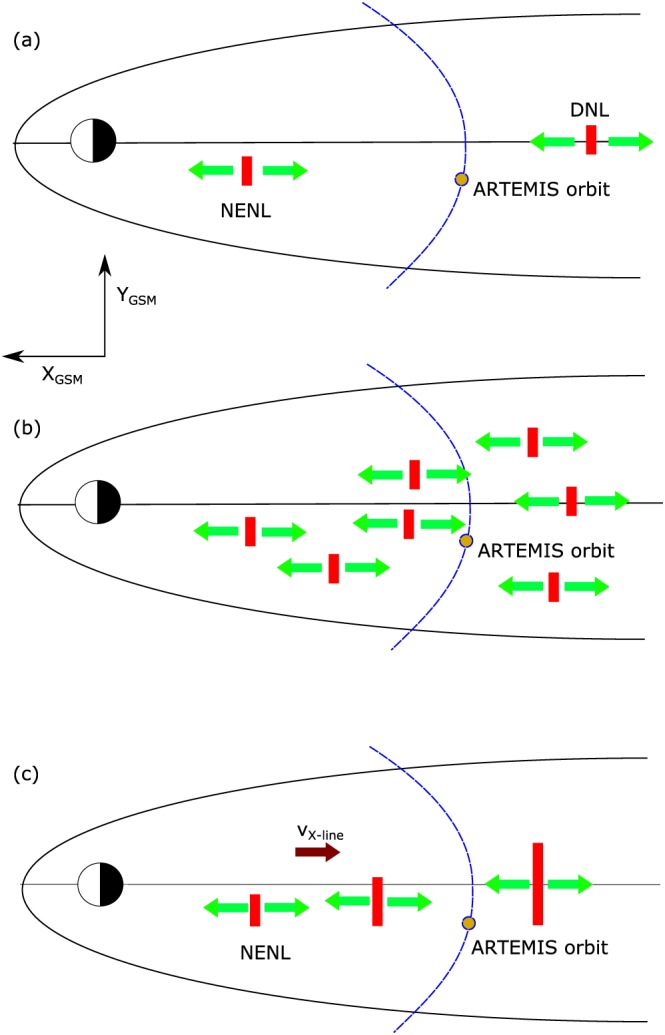
Three possibilities for the X‐line source of tailward and earthward flows observed with ARTEMIS. From top to bottom, (a) the classical NENL‐DNL concept, (b) patchy reconnection with a gradual decrease of the dawn‐dusk asymmetry with downtail distance, and (c) a tailward moving X‐line moving beyond ARTEMIS orbit.

We see three possibilities for the X‐line source of earthward and tailward flows observed near lunar orbit (see Figure 10): (a) the classical dual near‐Earth neutral line (NENL) and distant neutral line (DNL) concept (Baker et al., 1996; Hones, 1977; McPherron, 1970); (b) patchy reconnection over a broad range in the magnetotail, as suggested by recent simulations (e.g., Wiltberger et al., 2015); (c) a tailward moving near‐Earth X‐line (Alexandrova et al., 2015; Maezawa & Hori, 1998; Oka et al., 2011; Russell & McPherron, 1973), which is responsible for both, earthward and tailward flows, depending on its dynamic location relative to ARTEMIS' orbit.

The statistical tendency of tailward convective flows to appear in the dusk sector indicates that X‐lines form preferentially in the premidnight sector earthward of *X*_GSM_≈−60*R*_*E*_, while the lack of a clear asymmetry in earthward convective flows for weak substorms indicates that X‐lines beyond *X*_GSM_≈−60*R*_*E*_ are evenly active in both sectors. When substorms evolve beyond AL <− 400 nT, the accumulation of flux transported to the near‐Earth region forces the conditions for reconnection onset to move farther downtail, which is in line with concept (c) of a tailward retreating X‐line. Therewith, the asymmetric behavior of the reconnection onset conditions progresses tailward as well—beyond −60 *R*_*E*_. However, our results can also be interpreted within concept (b), as the asymmetry condition of the reconnection location spreads throughout the magnetotail for AL <− 400 nT, allowing reconnection to occur preferentially in the dusk sector at multiple locations during strong substorms. Concept (a) with a near‐Earth X‐line primarily in the dusk sector and a symmetrically extended distant neutral line, seems to be only applicable during weak substorms.

## Erratum

In the originally published version of this article, funding information was omitted from the Acknowledgments. The funding information has been updated to include reference to NSF/GEM grant 1503097, and the present version may be considered the authoritative version of record.
